# Enabling personal recovery from fibromyalgia – theoretical rationale, content and meaning of a person-centred, recovery-oriented programme

**DOI:** 10.1186/s12913-021-06295-6

**Published:** 2021-04-14

**Authors:** Anne Marit Mengshoel, Åse Skarbø, Elisabeth Hasselknippe, Tamara Petterson, Nina Linnea Brandsar, Ellen Askmann, Ragnhild Ildstad, Lena Løseth, Merja Helena Sallinen

**Affiliations:** 1 Department of Interdisciplinary Health Sciences, Faculty of Medicine, Institute of Health and Society, University of Oslo, Box 1089, Blindern, 0317 Oslo, Norway; 2 Hospital for Rheumatic Diseases, Margrethe Grundtvigsvei 6, 2609 Lillehammer, Norway; 3 Faculty of Health and Welfare, Satakunta University of Applied Sciences, Pori, Finland

**Keywords:** Fibromyalgia, Patient-centeredness, Patient education, Coproduced knowledge, Personal experiential recovery process

## Abstract

**Background:**

Fibromyalgia (FM) is a contested, chronic widespread pain syndrome on which recommended therapies have short-lasting, moderate effects. Nevertheless, some patients become symptom-free, and their recovery experiences inspired us to develop a patient-centred recovery-oriented programme (PROP) delivered in a group format. Presently, we describe the theoretical rationale, purpose and content of the PROP, and its meanings for clinicians and patients.

**Methods:**

A multidisciplinary clinical team, a leader of a rehabilitation unit, and two researchers coproduced the PROP. Five full-day seminars were arranged to bridge research and clinical experiences. Qualitative studies about patients’ illness and recovery experiences and questions by researchers facilitated reflections on clinical experiences. The meaning of the PROP was examined using focus group and individual interviews with patients and clinicians immediately after completing the course and after 1–1.5 years.

**Results:**

The biopsychosocial model displays the research evidence across biological, mental and social impacts of FM, justifying that life stress can be an illness-maintaining factor in FM. The content addresses enabling patients to heal their own life and self by modifying life stress. Patients engage in making sense of the relationship between FM, themselves, and life through exploring, discovering and creating appropriate solutions for their daily social life. The PROP reduced uncertainties and brought a positive attitude and hope to the groups. After 1 year, patients are still engaged in recovery work, experience more good days, and maintain hope for further recovery. By sharing and reflecting on clinical experiences, a unified clinical team was established that continues to develop their competency.

**Conclusion:**

To our knowledge, the PROP is the first programme for patients with FM that results from a process of coproducing knowledge, is based on explicit theoretical rationale, and facilitates a personal experiential recovery process. PROP is found to be meaningful and to work by patients and clinicians.

**Supplementary Information:**

The online version contains supplementary material available at 10.1186/s12913-021-06295-6.

## Background

Fibromyalgia (FM) is a prevalent condition characterised by long-term widespread musculoskeletal pain associated with excessive fatigue and non-restorative sleep [1, 2]. Patients also report several other complaints such as headache, irritable bowel, depression and cognitive problems [3]. The aetiology of FM is unknown, but its pathogenesis is ascribed to amplification of pain impulses within the central nervous system [4]. Additionally, findings of neuroendocrine perturbations suggest dysfunctional responses to stress [5]. FM has considerable social impacts as symptoms disturb ordinary functioning at home, work and leisure time, as well as social relationships [6].

FM cannot be verified by any blood or radiological measures [3]; it is based on patients’ symptom reports of long-lasting widespread musculoskeletal pain, fatigue, non-restorative sleep, concentration and memory problems, headache, depression and abdominal pain/cramps [7]. During the clinical diagnostic process, patients are examined to rule out other diseases such as neurological, metabolic or rheumatic inflammatory diseases, which is why patients are often referred to various medical specialists [8]. A survey in European countries, South Korea and Mexico showed that reaching a diagnosis of FM may take years [9]. In this process, patients experience that health professionals (HPs) consider their symptoms to be imaginary or psychological, and this continues even after being diagnosed. This highlights that the FM diagnosis is contested and does not necessarily legitimate patients as being sick [8]. In addition, it is not known how the biological abnormalities can be reversed; therefore, treatments aim at relieving symptoms, educating patients how to cope with them and promoting a healthy life style.

In 2017, the European League Against Rheumatism (EULAR) published evidence-based recommendations for the clinical management of FM [10]. The guidelines build on systematic reviews of effect studies and were authored by a group of 18 members, including patients, researchers and HPs with background in various medical specialties and nursing. The group underlined the importance of a prompt diagnosis and informing patients about the condition. For management, they primarily recommended non-pharmacological therapies. Their first choice was conditioning exercise as they had found strong evidence that exercise relieved pain and improved physical functioning. Although the group found their other recommendations to be based on weak evidence, cognitive behavioural therapy was advocated for patients with low mood and inadequate coping strategies, and more generally, multicomponent therapies, acupuncture, hydrotherapy, meditative movement therapies (qigong, yoga, tai chi) were proposed. Duloxetine, pregabalin and tramadol were authorised by the group for patients with severe pain, and amitriptyline, cyclobenzaprine and pregabalin for those with sleep problems. However, both non-pharmacological and pharmacological therapies show only moderate, short-lasting symptom-relieving effects [11]. Thus, there is an urgent need to rethink and develop new interventions.

In the present study, a multidisciplinary team of HPs, a leader of a rehabilitation unit and researchers collaborated in developing a new patient education programme. The HPs had several years of experience of delivering a patient education programme in line with the EULAR guidelines, but they were uncertain as to whether the effects anticipated by the EULAR group were met and of their particular roles in the existing programme. The leader experienced that the programme lacked a shared vision and understanding of FM, and she questioned whether the profession-driven education in symptom management and healthy lifestyle met its claims of being person-centred. For their part, the researchers had for years studied patients’ illness experiences [12–14] and former patients’ experiences of recovering from FM [15–17]. Their opinion was that knowledge about patients’ illness and recovery experiences can inform HPs to better understand and meet patients’ needs. With the various critical questions as a backdrop, we initiated a project in collaboration with clinicians to improve the existing patient education program. The project was inspired by the idea of co-producing knowledge [18], aiming at closing a theory-practice gap by bridging research-evidence and clinical context-specific knowledge, i.e. practice-evidence [19]. This collaboration process resulted in a quite different patient education programme than the original one. Our main purpose presently is to describe this new patient education programme.

### Patients’ illness and recovery experiences

Patients were not involved in developing the new patient education programme. Therefore, in order to take the patient perspective into consideration and to meet our goal of developing a patient-centred programme, we actively used and discussed qualitative studies about patients’ illness and recovery experiences during our meetings. Some authors call FM an illness without disease [20] and express concern of medicalising patients’ illness experiences [21]. Illness connotes a subject’s experiences of how disease and its consequences are perceived [22, 23]. Such experiences can include alienation from own body, disrupted ordinary life, removal from social roles and obligations, and disturbed relationships with family, work colleagues, and friends [24]. Thus, illness includes several negative experiences, which are construed through reciprocal interactions between an individual, her/his social life and contexts [24]. It is essential to make sense of the new situation and find practical solutions for managing the problems illness creates socially [25]. Therefore, recovering from illness includes making sense of what is happening and discovering how to recreate a dignified life within the limitations set by the disease [26]. Opposite to illness is wellness, which may alternate between being in the foreground or background [27]. Such shifts imply that patients must work on their illness experiences in order to keep wellness in the forefront. Hope fuels such recovery efforts and progress motivates further actions [28]. In contrast to ‘repairing’ a disease, recovering from an illness comprises patients’ efforts of coming to terms with or overcoming illness; in other words, healing oneself and one’s social life.

In FM, multiple unfamiliar bodily sensations separate the person from the body and the daily life they used to know; their own body has become a stranger and an object for scrutiny that disturbs social life in various ways [29]. For example, work is complicated by memory and concentration problems [30] and exercise by post-exertional malaise [31]. The severity of symptoms fluctuates, often without any apparent reason [32]. Thus, the body can no longer be taken for granted and trusted, and this brings about worry and uncertainty [33]. Patients carefully monitor their body and take action to avoid intensification of symptoms [34] by finding out what triggers symptoms and by adjusting their behaviour and life accordingly [32]. Activities are planned on a day-to-day basis, and plans and everyday routines are often disrupted. As put by McMahon et al. [35], life is ‘governed by pain’. The distress is further accentuated by inability to fulfil social obligations, social roles and relationships [34].

It is stressful to be diagnosed with FM because many HPs question the ‘realness’ of symptoms [33] and patients feel they are overlooked and not taken seriously [12]. They can be seen as hypochondriacs and not doing their best to recover [8]. The patients mourn over their losses and wish to get their prior lives back [36]. Former patients’ recovery experiences portray a long, strenuous process of making sense of how bodily sensations relate to daily life in order to avoid intensifying symptoms [15, 17, 37–39]. Through a rather mundane daily trial and error process to find appropriate solutions, they developed personal competence that enabled them to slowly overcome FM [37]. The progress was hardly noticeable, but over time they recognised improvements that created hope for a better future [17]. They interpreted symptom flares as a warning of accumulation of too much stress over time, which helped them to make appropriate adjustments [15]. Wentz [38] and Sallinen et al. [39] also suggest that modifying stress is important for recovery. In sum, these studies suggest that patients may recover from FM by acting on their illness experiences by considering them in light of stress intolerance.

With the above theoretical knowledge as an inspiration, we set out to develop a person-centred patient education programme that ended up to become a patient-centred, recovery-oriented programme (PROP) tailored to patients’ experiences. The purpose of the present paper is to describe the theoretical rationale, purpose and contents of the new programme, and its’ meaning to clinicians and patients.

## Methods

### Design and ethics

A participatory action research design was applied [40]. This design includes a spiralling process of alternating between critical reflections, actions, systematic data collection and analysis, trying out if solutions work in practice, and bringing new insights into the further development process. A multidisciplinary clinical team, an administrative and clinical professional leader, and two researchers (AMM, MHS) collaborated in developing a clinically usable programme [18, 41]. The process was implemented in a flexible but systematic manner. The HPs and patients were given oral and written information about the purpose of the study and the right to withdraw without any consequences for future career or treatment at the hospital. Informed written consents were obtained and the Norwegian Data Inspectorate for Research approved the study (no. 2018/57956/3/EPA).

### Study context and participants

Since the 1990s, a multidisciplinary team at the Hospital for Rheumatic Diseases in Lillehammer, Norway, has offered a patient education programme for ambulatory and hospitalised patients with FM. Currently, a team of 12 to 14 HPs with background in medicine, nursing, nutrition, occupational therapy, physiotherapy, psychology, and social work was involved in the development of a new programme. Most of them have worked for many years with chronic pain patients at the hospital or at other clinics, and several of them are educated in counselling pedagogy. Two researchers with prior clinical experience as physiotherapists in mental health, primary health care, and rheumatology guided the work. For years, they have been employed fulltime at universities in Norway and Finland, responsible for lecturing and supervising master’s and PhD students from various professional backgrounds in performing qualitative and quantitative studies broadly within the field of rehabilitation. In their own research, however, they maintain a specific interest in FM. One researcher (AMM) was familiar with the HPs and the context beforehand while the other researcher (MHS) had an outsider perspective.

### Coproducing a personal recovery-oriented programme

Coproduction of knowledge entails a new approach for knowledge development [41] based on the view that together, people with various perspectives and knowledge who collaborate on equal terms can produce new and better knowledge to solve clinical challenges. Thus, coproducing knowledge aims at developing a collective wisdom, and it was presumed that this would result in a practice for which HPs have ownership [42]. Patient education aims at teaching patients to live well despite illness, and therefore, research evidence about patients’ illness and recovery experiences was actively brought into the coproducing process.

Five full-day workshops were arranged at the hospital over a four-month period. The workshops served as an arena for the participants to express ideas, share experiences, and search for further insights. Prior to each meeting, the researchers distributed two or three qualitative papers addressing patients’ experiences of diagnosis, treatment, living with FM, or personal experiential recovery process. In the workshops, the clinicians compared their experiences with those reported by patients, first in a plenary brainstorming session and then in small groups of 5 to 7 participants to dwell in more depth on issues raised during the plenary discussions. The researchers moderated the discussions and asked questions to clarify what was said [43].

### Data collection and analysis

During the plenary discussions, one of the researchers took notes, and the small group discussions were tape-recorded. Immediately after the workshop, the researchers read the field notes, listened to the audio-recordings, and summed up the reflections. In this process, practice and research evidence were merged to come to an overall understanding about what is at stake for patients with FM, how patients can engage in their own recovery process, and how HPs can provide appropriate help. Theoretical interpretations of disease, illness, recovery, and health helped us to make sense of and systematise the data. Summaries and preliminary interpretations were developed and presented orally at the next meeting. This served to validate the researchers’ interpretations and often led to further reflections on the topic. For the last workshop, a draft of the PROP’s rationale and contents was displayed by biopsychosocial and resource-oriented recovery models, respectively, and the HPs concretised the PROP’s content.

### Evaluation of the programme's feasibility

#### Focus group and individual interviews

In order to find out how the programme was perceived and if adjustments in the programme were needed, both patients and HPs were interviewed. The feasibility of the PROP was addressed by focus group and individual interviews with HPs who delivered the programme and with patients who had participated in the programme. All HPs (*n* = 8) who delivered the programme, were interviewed in a focus group by the first author (female) just after the first group of patients had completed the course. The interview addressed three pre-planned themes; the meaning, relevance and delivery of PROP. At the time of the first interviews, everyone had participated in developing the PROP, while after 1 year, two team members with experience in delivering but not developing the PROP were also interviewed. The HPs delivering the PROP had background in medicine, nutrition, occupational therapy, physiotherapy, psychology and social work (*n* = 8). After 1 year, the HPs were interviewed individually by the last author and a research assistant (both females) inspired by narrative methods aiming to understand their experiences of delivering the PROP as a process evolving over time [44]. They were asked to tell about their experiences working with patients with FM and to deliver the PROP, and throughout this story-telling they addressed how it was like before, how it had changed over time, and how it was like today (for details, see supplementary material).

Immediately after completing the course, all eight patients from the first PROP group participated in a focus group interview lead by the first author that did not know the patients at beforehand. But the participants knew the purpose of the study and why they were interviewed. In addition, four from the two first groups were interviewed individually. In these interviews, the following pre-planned themes were addressed; relevance and meaningfulness of the programme, as well as the content, structure and delivery of the PROP. About 1–1.5 years after the programme, in order to explore long-term possible significance of the PROP, we randomly selected three patients from each of the four groups (*n* = 41) for individual telephone interviews conducted by the first author. Nine female and two male patients, mostly in their 30s and 40s, volunteered for individual telephone interviews; three of them also participated in the first interviews. Everyone had experienced symptoms of various severity for years. Some were diagnosed several years ago and others recently. In the follow-up interview, they were asked to tell freely about a process from what had happened before the PROP, after it, and what their situation was at present in line with narrative interviewing [44]. The purpose was to learn about their personal illness and recovery process to find out if eventually the PROP had any impact for their process. The interview guides for the focus group interviews and the individual interviews are shown in the supplementary material. The interviews with HPs and patients were tape-recorded and transcribed verbatim. Both senior researchers listened to the recordings and discussed the meanings of them afterwards. The text about the PROP and its relevance was extracted, coded and sorted into categories according to thematic analysis [45]. However, presently only the overarching preliminary findings are given in order to provide an argument for the feasibility of the PROP in our setting, and therefore, why it was worthwhile to provide a detailed description of the PROP.

## Results

### Towards a personal recovery-oriented programme

#### Why a programme tailored to modify life stress

The ‘old’ content of the programme was profession-driven and included education about FM, pain mechanisms, nutrition, social rights and security systems, conditioning exercise, activity regulation, mindfulness and acceptance-based psychological approaches. However, it was unclear how the various components corresponded to FM and how the components were related to each other. In other words, an explicit theoretical understanding of FM and an overarching purpose of the programme were lacking. Thus, we had to provide a theoretical justification for the PROP and link its contents to an explicit purpose.

**Fig. 1:**
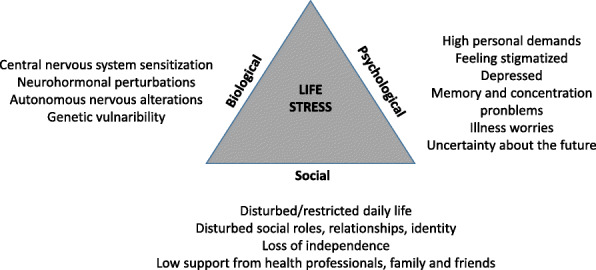
An overview of our understanding of the complexity of fibromyalgia

During the coproducing process, the HPs repeatedly referred to their work as aligning with a biopsychosocial understanding, often applying concepts of body function, activity and participation in accordance with the WHO’s International Classification of Health and Functioning, ICF [46]. The ICF is based on a biopsychosocial understanding, illustrating that health is constituted through complex, dynamic, interactions between biological, psychological and social dimensions [47]. Accordingly, FM can be interpreted as being wound up by interactive processes between biology, psychology, and social problems (Fig. 1), indicating that such a vicious circle can be acted upon in several ways.

**Fig. 2:**
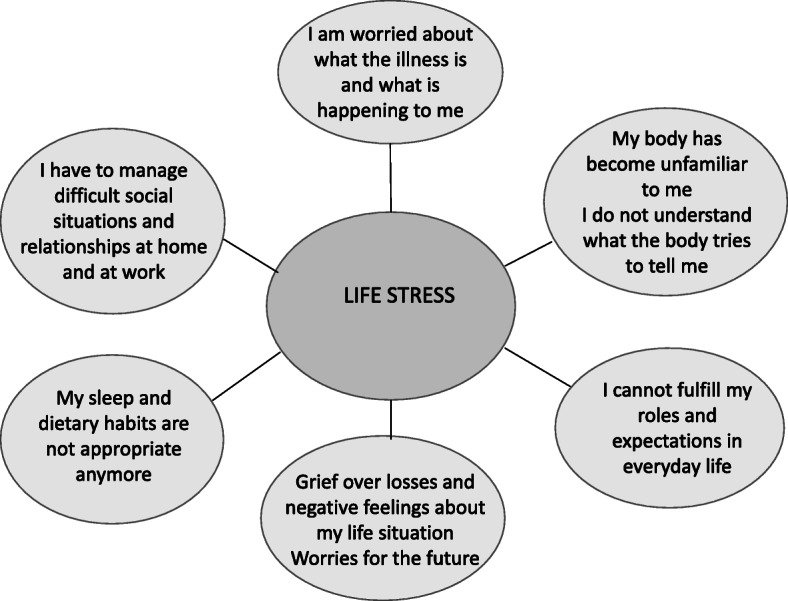
Issues addressed by the patient-centered recovery-oriented programme for patients with fibromyalgia

Much research suggests that for a person with FM, it is distressing that FM is an unexplainable and unpredictable illness which interferes with participation and relationships in social life while being, at the same time, a socially contested illness [32]. Furthermore, this stress can be accentuated through social stressors such as high mental and physical demands, exhaustive care giving, familial conflicts, and job dissatisfaction [48]. A person’s worries, catastrophic thinking, low self-efficacy or depression may perpetuate the perception of stress [48]. Biologically, stress is understood as a threat to the body’s homeostasis which activates a ‘fight and flight’ response [49]. The biological stress response system is mobilised through activation of the hypothalamic-pituitary-adrenal (HPA) axis and the autonomous nervous system [50]. In FM, altered responses of the autonomous nervous system to stressors such as exercise [51–53], ice water [54], and tilting [55] are reported, as well as blunted cortisol responses in the HPA axis [5, 48, 56]. It is also reported that premorbid stress in the form of an intensive lifestyle may lead to the onset of FM [57], and symptoms are intensified by overdoing, mental and physical stress [58, 59]. We therefore argue that life stress, as a sum of stress related to FM and generally in life, may hinder recovery from FM. This understanding underpins why the PROP is tailored to modify life stress. Our starting point is that it is crucial to make sense of FM and how it intertwines with daily life, and thereafter, modify stress by taking practical steps to adjust life and by challenging unhelpful beliefs and attitudes (Fig. 2).

#### What to address and how to deliver the programme

##### Uncertainties related to context and practice

Reading about patients’ experiences of the diagnostic process and the FM label gave rise to reflections among the HPs about the disputes related to the diagnosis within medicine and society in general. The HPs felt they had to defend their delivery of services for this patient group against the scepticism towards the ‘realness’ of FM and health authorities’ recommendations that patients with FM should be helped in primary health care. However, the HPs recounted that several patients had told them that they had often been ignored, neglected or treated badly by professionals in primary health and social services. Patients’ prior negative experiences influenced the HPs’ consultations with patients, and they needed time to build good relationships with their patients. Therefore, the clinicians watched their steps; as one clinician put it: *‘I do not behave like this in encounters with other patients. I just act as a pussy-cat’*. Thus, in the PROP, it became important at an early stage to address the patients’ former experiences with HPs and services to establish a trustful relationship.

The HPs felt updated by participation in conferences and following evidence-based management guidelines. At the same time, they felt that their knowledge fell short. The professionals trusted the research-based recommendations but were uncertain of whether they achieved symptom relief and improved physical functioning as outlined in the EULAR guidelines [10]. A dilemma was that the patients did not always follow their recommendations, and the HPs were uncertain of how to best motivate patients to adhere to them. Nevertheless, the team felt that they made a difference to patients, and perceived that patients found it important to share their experiences and learn from peers and HPs. However, it was difficult to explicate why and how this played a role. In the PROP, the themes and issues are linked to life stress (Figs. 1 and 2) and nested in an understanding of recovery as a personal experiential recovery process, as outlined earlier. Sharing experiences was carried forward from the prior programme and became an important ingredient in the PROP to enable patients to find out how to get better.

##### Enabling patients to practise recovery work

The non-medical HPs experienced that in order to motivate patients to engage in their approaches, they had to stop patients’ ‘*doctor-shopping*’ for a cure, change illness talk and helplessness into a more positive talk about possibilities, and convince patients that they could get better through their own efforts. Thus, the non-medical HPs considered that it was crucial for their work that a physician confirmed the diagnosis beforehand, offered a thorough medical explanation of FM, and a notion of why it was important for patients to ‘*come to grips*’ with their own situation. In other words, the physician had to set a starting point by explaining why recovery can be achieved through one’s own efforts.

The former patient education programme was person-centred in terms of respecting autonomy and formulating rehabilitation goals in line with individuals’ particular wishes and choose what was most appropriate for them from a ‘*tool-box’* presented by the HPs. The PROP takes this further, as person-centredness is now connected to engaging individuals in their recovery process by acting upon own experiences. HPs and patients collaborate in making sense of individuals’ illness experiences, and together, they discover how individuals can protect themselves against stress, modify life stress in their own life situation, and rebuild tolerance to life stress. It is crucial to promote individuals’ resources and engagement in exploring, facilitate learning from their own and others’ experiences and adjust social practices and thinking to remake a tolerable, still meaningful life. Corbin and Strauss [60] outline that people with long-lasting illness have to perform three interacting lines of recovery work: illness trajectory work, everyday life work, and biographical work. These lines of work together with the HPs competencies determined the issues to be addressed in the PROP (Fig. 2).

In order to make sense of illness experiences, discover possible solutions and try out and acquire new experiences, clinicians provide introductory information about predefined issues for further reflections in the group (Table 1). To facilitate reflections, HPs use practical exercises in plenary or small groups inspired by confluent pedagogy [61], exemplified in Table 1. The explorative and reflexive process refers both to the participants’ internal conditions such as attitudes, values, hopes, life expectations, power, and feeling of control and connectivity, and to external conditions such as habits and routines of daily life, stress management techniques, social support and policies. To maintain motivation to carry on, patients learn to recognise and acknowledge their progress. The fundamental values underpinning the PROP are shown in Table 2. Each group included 8 to 12 patients, and the courses for each group were delivered as eight full-day seminars (six hours a day including lunch arranged in intervals of 2 to 4 weeks. The seminars were led by two clinicians.

**Table 1:** Content of the person-centred, recovery-oriented programme for patients with fibromyalgia

Recovery modules	Themes addressed	Examples of exercises to facilitate awareness and acting on own experiences during the course
Day 1 Opening and introductory information	*Physician and physiotherapist:* FM and plausible causes, pathogenesis and treatments Significance of physical activity	**Plenary group discussions:** Lectures by health professionals and one lecture by a user representative set the route for discussions among the participants, the health professionals and the whole group and afterwards in groups of 2–3 patients **Individual exercises and a written diary:** Develop an awareness of own life situation by writing down own reflections on issues brought up during the day. For example; ‘life stress is for me …’ Describe three priorities you will work on further. Develop an awareness of daily life habits and routines by mapping activities during a day, thereafter, describe what tasks you must do, want to do, do not like or need to do Develop an awareness about energy consumption by recognizing what drains or increases energy in daily life Develop an awareness about preferred self; who am I, what values are important to me, who do I want to be Develop awareness of own progression by writing down discoveries and reflections at the end of each course day **Discussions with 1–2 peers:** Share with peers challenges identified through individual exercises and listen to their reflections about it for example during ‘walk and talk’ **Practising various forms of physical activities guided by the physiotherapist such as:** Various outdoor activities, for example Nordic Walking with sticks Activities in warm water pool Relaxation techniques Medical yoga Restoring exercises during a day
Day 2 Stress and illness	*Physician:* Medical understanding of FM and pharmacological treatments *Psychologist and occupational therapist:* What is stress and what does it do? Accumulation of stress during a day, identify and prioritize activities	
Day 3 Energy	*Psychologist:* Why is sleep important, how does sleep relate to stress, and how can sleep disturbances be managed? *Nutritionist:* The role of diet and eating routines for energy	
Day 4 Identity, life values and communication	*Social worker and psychologist:* How can identity, social roles, relationships to others, and own values be maintained despite changed prerequisites?	
Day 5 Work and health	*Occupational therapist and social worker:* Ergonomics and practical adjustments Economical rights and support, duties and possibilities	
Day 6 Body awareness	*Physician and physiotherapist:* Intelligent body – what does the body try to tell? *Psychologist:* Symptoms as a resource for acting on own experiences Cognitive functioning and management	
Day 7 Being in recovery	*Programme leaders:* Turning points and recognizing progress Setting and revising attainable goals	
Day 8 Way ahead	*Social worker and programme leaders:* To keep focus and motivation Finding supporters

**Table 2:** The fundamental values underpinning the patient-centered, recovery-oriented programme for patients with fibromyalgia

1. A belief in a person’s resources and strengths to undertake personal recovery work 2. A personal recovery work is self-determined and self-directed 3. A person can recover by learning from peers’ and HPs’ knowledge 4. A personal recovery work aims to remake a well life for oneself 5. A personal experiential recovery process takes time and hope is essential to endure 6. HPs’ role is to coach and support patients’ process of learning and recovering	

### Relevance and benefit of personal recovery-oriented programme

During the project, the PROP was delivered for four groups of patients; comprising in total 41 patients. Their characteristics are shown in Table 3. There was a great heterogeneity among the participants with respect to age, educational level, employment status, duration of pain, and time since diagnosis. Most of the participants, however, were females, and the FM severity score frequently was graded as very severe. Only one patient did not complete the PROP. The reason was that she was offered another service she expected to be helpful. She volunteered for an interview after one year.

**Table 3:** Characteristics of the patients participating in the patient-centred recovery-oriented programme

Patients ***n*** = 41	Mean (standard deviation)	Minimum – maximum score	Percent of ***n*** = 41
Age in years	45 (11)	20–66	
Women			81%
Married/cohibitant			81%
Symptom duration, in years	11 (11)	0,6–42	
Time since diagnosis, in years	4,4 (8)	0,1–33	
Fibromyalgia Survey Questionnaire ^a^ 0 = best 31 = worst)	23.2 (3.7)	11–30	
Educational level			
• ≤12 years			54%
• Completed high school			20%
• College or university			26%
Employment status			
• Full- or parttime employed			29%
• Partly on sickleave			24%
• Full sickleave			44%
• Job seeker			2%

^a^Assessment of fibromyalgia severity: none (0–3), mild (4–7), moderate (8–11), severe (12–19), very severe (20–31)

#### The users’ immediate experiences

##### PROP is found meaningful by patients and HPs

The theoretical rationale for tailoring the PROP to modify life stress was found meaningful by both the patients and the HPs, and the content was found relevant and clearly nested in the overarching purpose. The patients felt acknowledged by the HPs and trusted their skills. The HPs’ presentations were informative; overall the patients appreciated the clinicians’ ways of moderating discussions and exercises for facilitating reflections in the group. The HPs’ lectures and reflections together with peers provided new insights that helped patients to discover and try out possible solutions for their lives, which in turn created hope that recovery was possible. Some patients disliked exchanging experiences with peers. For example, one patient did not like the exercise where they expressed a concrete problem, and then during an outdoor walk, two peers discussed solutions for the problem while the one ‘owning’ the problem walked a few steps ahead and listened to their reflections. Others found such exercises valuable as it distanced them from their own challenges. They did not always agree with the suggested solutions, but found it helpful to rethink them.

##### Downscaling ambitions

During the focus group, some patients expressed that it was impossible to do anything about their life situation. In particular, this was expressed by those with high burdens of care for disabled children or old relatives. Thus, it was later emphasised by the HPs that even small changes in life situations could make a difference. In other words, it was not only about prioritising activities and routines, but also discovering how small adjustments could be made within particular habitual daily situations; for example, by taking small breaks or alternate between energy-demanding and less demanding activities.

For their part, the HPs reported that some patients did not join the exercise classes, especially those arranged in the gym. In an individual interview, a patient told that she hated gym at school, but during the PROP, she discovered that her headache disappeared during an outdoor walk which incorporated body awareness and relaxation exercises. Thus, she felt that this, and not training, was right for her (a practice still sustained after 1.5 years). This reminded us that stress can be modified by physical activity, body awareness, relaxation, meditative exercises as well as nature. The HPs agreed to consistently use the term physical activity instead of training in the following courses. Another issue highlighted by the HPs was to change wordings from ‘*understood*’ into ‘*learned*’, and instead of asking ‘*what have you done*’, to ask ‘*what have you discovered*’. They also decided to write a ‘*blog*’ with some keywords related to the course day to share and discuss with the rest of the team at the end of the day. Both HPs and patients found the course days too long; accordingly, in the following courses they were shortened from seven to six hours.

#### The users’ long-term experiences

##### Patients continue their recovery work

Even though the participants took part in the PROP 1–1.5 years ago, they still found what they had learned meaningful for their current and future process. One recently diagnosed man said that ‘*It was like winning a lottery to be referred to this programme*’ while others felt sorry that they had not learned about it before. They had also recommended the PROP to their family physicians and acquaintances.

Initially, the patients might have believed that it was impossible to do anything about their life situation. However, over the last 1–1.5 years, they had become attentive to the relationship between their body, themselves and social life. They had changed their daily routines and adjusted daily situations. For example, a patient had got respite care for a disabled child and was able to spend more time with her husband and the other child, while others enjoyed coffee with their older relatives instead of ‘*hurrying around to manage everything for them*’. Importantly, in the interviews, the patients connected themselves to the present and future instead of ruminating on the past. They expressed confidence in their own healing capacity and believed in further recovery. They still experienced flares, however. But, compared to previously, they experienced more good days when they were more or less symptom-free, and they now resisted overdoing things on good days to ‘*avoid being punished*’ the next day. They found it helpful to take small breaks during the day, do meditative exercises or walk in the forest. Some felt also more energetic due to regular physical activity, regular meal times and increased intake of healthy food items. Overall, except for one participant who only attended two course days, the narratives expressed being in recovery.

##### A professionally confident and unified team

The shared purpose of targeting life stress had made it easier for the HPs to define and adjust their practice, and it enabled them to see how the various pieces of the PROP contributed to its wholeness. One said that she had previously felt as ‘a guest lecturer’, but now everyone expressed ownership of the PROP and perceived themselves as important contributors in the team. The team members recognised and valued each other’s perspectives, and they had continued to learn from their experiences through 15-min meetings at the end of each course day. This endeavour strengthened their ownership of the PROP; in the words of one HP, ‘*in this process my own professional perspective has become clearer to me*’.

Previously, the patient education programme addressed healthy life style and psychological processes of coping with symptoms. In the PROP, the main focus is on social life: daily tasks, thoughts about one’s own real-life situations, social roles and identities. In this way, the complexity of FM became concretised for both patients and HPs, and as one of the HPs put it, ‘*more ordinary and easy to understand*’. The HPs found it exciting to encourage patients to discover their own possibilities for recovering, and they had noticed that a positive attitude and hope were brought into the groups. In contrast to initial debates about unmotivated patients and repetitive illness talks, nobody mentioned this anymore.

Facilitating a patient’s explorative process implied that the HPs no longer needed to know what was the right thing for the patients to do; rather, they needed to be more open-minded and collaborate with patients to discover possible solutions. Actually, this meant a change in therapeutic attitude; as one HP stated, ‘*we are educated to solve problems for patients, not to encourage patients to find them out for themselves*’. However, it was a balancing act for HPs to know ‘*what was the right time and which button to push*’ in order to facilitate the explorative process. The group dynamics could also be challenging; for example, how to slow down talkative patients and engage silent ones, and how to avoid one patient taking a lead role over others. It was also puzzling to recognise and applaud small events that signified breakthroughs. A vocabulary in line with personal experiential recovery was incorporated in HPs’ narrations, including words such as meaning, turning points, hope, strengths, and resources. They felt confident about their role in the team and contribution to the PROP, and their engagement and enthusiasm were considered to matter to the patients as well.

## Discussion

A new health service for patients with FM was developed based on the assumption that life stress hinders recovery, and therefore the content was tailored to enable patients to engage in healing their social daily lives and selves by modifying life stress. The service and its use were found meaningful and to work by patients and clinicians.

In rheumatology, it is a long tradition to educate patients with arthritis to manage their disease with drugs, do exercises to prevent joint stiffness and malfunction, and accommodate to healthy life style regimens [62]. Unfortunately, for patients with FM, there are no known effective relieving and preventing management methods, and both quantitative and qualitative research suggests that patients’ lives are ruled by illness. There are biological alterations and mental problems related to FM that can possibly be alleviated by drugs, but here, we tailored our PROP to the person-in-social-life context to enhance patients’ own engagement in acting upon their own illness experiences. In this way, patients are supposed to adjust their habits, routines, and daily situations. To guide and make sense of such a process, we assumed that patients need an overarching vision as a guidance, currently to modify life stress. This is also justified through medical explanation and the programme’s content. The HPs’ knowledge and recognition of how stressful it is to live with FM and their active engagement in exploring patients’ experiences seem to help patients to understand and discover what to do in order to come to terms with and gradually overcome FM. For the HPs, the personal recovery process became concrete, or as expressed by one of them, it became ordinary by linking it to real life situations. As stated in a study of ‘burnout’ [63], people with illness have to re-habituate themselves to life. In this way, HPs consider patients not as victims of incurable illness, but as social beings with resources to recover, and this may bring a more positive attitude to the group and awaken hope that persists over time, as expressed by those interviewed. In turn, this may protect HPs against pessimism and burnout [64].

Today, the delivery of health services should be patient-centred, and this was also agreed upon by the HPs in the present study. Gluyas [65] argues that patient-centredness relies on the relationship between patient and health provider. She describes three types of relationships: a paternalistic relationship where the HP is the expert and determines what is the best treatment on behalf of the patient, an informative relationship where the HP informs the patient about illness and treatment options and expect the patient to make informed choices, and an interpretative relationship where HPs take the time to find out what is important for patients and help to sort out possible solutions in order to attain patients’ desired outcomes. The prior patient education programme was aligned to the second kind of relationship as the information provided to the patients was determined by the disciplinary perspectives of the HPs while patients were expected to make relevant choices based on the ‘*tool-box*’ of solutions given by the HPs. By applying research evidence to form a justification of the PROP, merging the research evidence and HPs’ positive experiences of exchanging patient experiences, and nesting the present practice in enhancing a personal experiential recovery process, the patient-HR relationship moved to the third type of relationship. Thereby, the patient-centredness in the PROP is reflected in its focus on patients’ individual experiences, resources and engagement [65].

In recent years, the concept of evidence-based practice has been criticised as it does not respond to critical questions and complexities in clinical practice [66, 67]. Our PROP is developed by merging research evidence, clinical experiences and professional knowledge, and as such, it is based on an amalgam of various knowledge sources. An unexpected output from reading and reflecting on qualitative papers of patients’ experiences was that these papers confirmed the uncertainties and complexities experienced by the HPs in clinical practice. Initially, the HPs felt uncertain about their competence, their disciplinary contribution, and how to meet patients with negative experiences from earlier clinical encounters. The reading and discussions certainly increased the team’s knowledge about FM, patients’ illness and recovery experiences, but perhaps even more importantly, they discovered that their personal uncertainties were shared by others and that they needed a shared vision and mind-set for their work. This unified the team, resulting in further collaboration in discussing what they had experienced in practice, which in turn developed their competency and practical skills.

According to the framework of developing complex interventions, it includes four stages: developing, feasibility/ piloting, evaluating, and implementing an intervention [68]. The present study’s main purpose was to report the results of developing and modelling an intervention. In a developing process based on coproduction of knowledge, the intervention depends on what kind of professional knowledge, clinical experiences and theoretical knowledge those involved actually have, as well as how they construct an intervention based on their knowledge. Certainly, other people may have arrived at other results than we did. The trustworthiness of the PROP depends on our success in providing a transparent description of the logical coherence between our understanding of FM, the purpose, the content and the way of delivering the PROP. This means that the text should provide adequate information enabling others to critically appraise and reflect on our work. It is not possible to describe everything in detail, but we think the description is transparent enough to enable researchers, clinicians, patients and stakeholders in other settings to appraise the PROP. Our theory-based programme may inspire others to create similar programmes in their context.

Usually, in order to be found effective, a causal linear relationship between an intervention and outcome measures has to be proved by randomised controlled trials. However, this project has not reached this stage. The feasibility part addresses human variability, meaning and values about what is right and wrong. Trustworthiness of this part can therefore be nested in what humans find meaningful and to be working in daily situations [68]. In this paper, it is a shortcoming that we have not reported a detailed analysis of the interviews, but only the overarching findings to show that the PROP worked adequately in our context. Nevertheless, this may not be so in other contexts. Thus, our findings cannot yet be generalised. The PROP needs to be tried out in other clinical settings as well. However, the theoretical foundation and the overarching purpose of the PROP, its’ content and educational approach seem promising. Nevertheless, we will underline that the PROP is also reliant on the HPs’ skills in for example establishing trustful relationships among the participants and in facilitating personal learning.

## Conclusion

To our knowledge, our PROP is the first programme for patients with FM resulting from a process of coproducing knowledge, as well as being based on explicit theoretical rationale. The PROP was found easy to understand, meaningful and to work by the users; importantly, it brought hope and engagement to those involved. Our work is in an early stage, but so far, the PROP has shown promising results. Hopefully, our PROP can inspire discussions, refinements and trials in other settings. If successful, it may end up in a future large-scale evaluation.

## Supplementary Information

**Additional file 1.** Interviewguides for focusgroups immediately after the course and individual interviews one year afterwards.

## Data Availability

Audiotapes and transcribed interviews are stored on Secured Data Server for Research at the University of Oslo, Norway. Data cannot be shared publicly in order to protect the participants’ privacy.

## Abbreviations

FM: Fibromyalgia
PROP: Person-centred, recovery-oriented programme
HP: Health professional
HPA-axis: Hypothalamic-pituitary-adrenal axis
