# Supplementary material for: Enabling personal recovery from fibromyalgia – theoretical rationale, content and meaning of a person-centred, recovery-oriented programme
Source: BMC Health Serv Res. 2021 Apr 14;21:339. doi: 10.1186/s12913-021-06295-6 (PMC8045361; doi:10.1186/s12913-021-06295-6)
Supplement: Supplementary file 1 — Additional file 1. Interviewguides for focusgroups immediately after the course and individual interviews one year afterwards. [file 12913_2021_6295_MOESM1_ESM.doc]

**INTERVIEWGUIDES FOR FOCUSGROUPS IMMEDIATE AFTER THE COURSE**

**To patients:**

**Introduction:**

You have participated in a new patient education programme. We do not think the programme is perfect, so we want to appraise and improve its content and ways of delivery. Thus, it is important for us to learn from your experiences to find out whether it was found meaningful and relevant for you.

Relevance of information:

- Have the programme given you relevant and applicable information (give examples)?
- Do you miss anything (give examples)?
- Are there anything you need more in-depth or should be excluded (give examples)?

Ways of delivering:

- Do the professionals present knowledge in an understandable way?
- Can you please give examples about good and poor ways?

Questions wanted to be addressed by the clinicians:

- What do you think about the way of addressing physical activity?
- What are your experiences about the exercises in gym and heated pool?

Reflections about the future:

- If you think about illness as sitting in a deep hole with a ladder you have to climb upwards to reach a safe ground under your feet; where are you now?
- Do you see a way to reach higher on the ladder in the future?
- What do you think about your further way?
- Are there anything you have learnt during the course that may bring you further?

**Of health professionals:**

- What are your general experiences of participating in the programme?
- Do you experience that the programme is coherent and relevant?
- What is working well (please give examples)?
- What is not working (please give examples)?

**FOLLOW-UP INDIVIDUAL INTERVIEWS AFTER 1-1.5 YEARS**

**Of patients:**

Introduction: Today, I like to learn from your experiences of living with fibromyalgia and what you have experienced until now, and how it is like for you today. Please feel free to tell your story in the way that suits you best, I will not interrupt you but ask questions afterwards.

*Can you please take me back to the very beginning before your symptoms started, when it started and what has happened afterwards?*

If not already told, ask:

- What do you think can be the reason why you became ill?
- What happened when you became diagnosed and afterwards?
- How are good and bad days like for you – meaning, management?
- Can you tell me about your treatment experiences – what has worked and not worked?
- Can you tell about what has happened after participating in our patient education programme and did anything in the programme become significant for you?
- What do you think about what has happened up until now, - what is good and what is bad?
- What do you think about your future?

**Of health professionals:**

Introduction: This time, I like to know about your experiences about working with fibromyalgia in general and within the new patient education programme.

Professionals’ perspective:

*Can you please tell me about your experiences of working with patients with fibromyalgia from when you started until now?*

If not already told, ask:

- What is your view on fibromyalgia?
- What is the core feature of the programme as you see it?
- What is different now from your perspective in comparison to earlier approaches?
- How is it like for you to work within the programme?
- If any, how has your role changed and can you describe your role in the team?
- What do you think works and not?
- What is the value of the developmental project?
- What can be transferred from this project to other areas of your work?

Patients’ perspective as interpreted by the professionals:

*Can you please tell me about what you think is important for patients with fibromyalgia?*

If not already told, ask:

- What is different to patients in comparison to the earlier approach?
- What is the feedback from the patients?
- What do you think patients benefit from?
- What are the outcomes that you can see?
